# Effects of herbal plant supplementation on rumen fermentation profiles and protozoan population *in vitro*

**DOI:** 10.14202/vetworld.2024.1139-1148

**Published:** 2024-05-17

**Authors:** Antonius Antonius, Roni Pazla, Ezi Masdia Putri, Muhammad Ichsan Alma’i, Erika Budiarti Laconi, Didid Diapari, Anuraga Jayanegara, Laily Rinda Ardani, Leni Marlina, Riris Delima Purba, Ruslan Abdul Gopar, Windu Negara, Sharli Asmairicen, Putut Suryo Negoro

**Affiliations:** 1 Research Center for Animal Husbandry, National Research and Innovation Agency (BRIN), Jl. Raya Jakarta Bogor Cibinong 16915, Indonesia; 2 Department of Animal Nutrition, Faculty of Animal Science, Andalas University, Jl. Limau Manis, Padang 25163, West Sumatra, Indonesia; 3 Edufarmers International Foundation, Government Relations Manager, Edu Farmers International Foundation, Jl. MT. Haryono Kav. 16, Jakarta 12810, Indonesia; 4 Department of Nutrition and Feed Technology, Faculty of Animal Science, IPB University, Jl. Agatis Kampus IPB Dramaga Bogor, 16680, Indonesia; 5 Research Center for Agroindustry, National Research and Innovation Agency (BRIN), Jl. Puspitek Tangerang Selatan, 15314, Indonesia

**Keywords:** feed digestibility, methane emissions, plant herbs, protozoa

## Abstract

**Background and Aim:**

In the livestock sector, particularly ruminants, an approach to minimize methane emissions can be carried out through a feeding strategy involving herbal plants containing bioactive compounds that can reduce protozoa and decrease methane gas emissions. The aim of this *in vitro* study was to analyze the effects of herbal plant supplementation on rumen fermentation, total gas, and methane production, *in vitro* dry matter digestibility (IVDMD), *in vitro* organic matter digestibility (IVOMD), and protozoa populations within the rumen.

**Materials and Methods:**

Two experiments were conducted in this study. Experiment 1 was conducted to determine the most promising herbal plants capable of increasing total gas production and reducing protozoan populations. Three potential herbals selected in Experiment 1 were continued in Experiment 2 as supplements in the palm kernel meal (PKM)-based ration (70% PKM + 30% herbal plants).

**Results:**

Experiment 1 revealed that *Eurycoma longifolia* (EL), *Cola acuminata* (CLA), and *Cassia alata* (CSA) were potential herbal candidates for enhancing total gas production and the percentages of IVDMD and IVOMD. In Experiment 2, supplementation with EL, CLA, and CSA significantly increased IVDMD from 62.84% to 70.15%, IVOMD from 61.61% to 53.18%, and NH_3_ from 13 mM to 17 mM, as well as reduced partial volatile fatty acids and total gas production. In addition, the methane gas and protozoan populations were reduced.

**Conclusion:**

The utilization of EL, CLA, and CSA effectively increased the production of total gas, IVDMD, and IVOMD while reducing methane gas protozoa populations in rumen fermentation compared with the control.

## Introduction

At present, humans are facing major environmental problems such as global warming. This issue is associated with several greenhouse gases, including chlorofluorocarbons (CFCs), methane (CH4), nitrogen oxide (N_2_O), and carbon dioxide (CO_2_), that accumulate in the atmosphere at high rates due to increased human activities [1]. The agricultural sector is one of the main contributors to the largest emissions of anthropogenic methane (CH_4_) in the world, with ruminants being the main producers. However, methane emissions are expected to increase by up to 30% by 2050 if they are not controlled immediately [2]. In addition, data from the FAO in 2017 indicate that the 100-year global warming potential for non-CO_2_ greenhouse gas (GHGs) from the livestock sector contributes approximately 15.6%–18.9% of all annual GHG emissions worldwide [3].

Methane gas (CH_4_) in ruminants results from the anaerobic fermentation of carbohydrates by methanogenic microorganisms (methane–producing bacteria). The gas is then released into the atmosphere by erupting (belching). Methane production can decrease energy utilization in the bodies of ruminants by 7%–12% [4]. The relationship between rumen protozoa and methane production has also been highlighted in several studies. Decreasing protozoa levels in the rumen leads to a decline in methane gas production [5]. This reduction in methane can be achieved by administering protozoan defaunators, such as saponins, which encourage the growth of bacteria that digest fiber. Tannins can reduce methane emissions by decreasing methanogens in the gastrointestinal rumen [6–8], hindering fiber digestion, thereby reducing H_2_ production. However, the quantity of methane (CH_4_) emitted by livestock is affected by factors such as type and amount of feed, genetics, and waste disposal methods [9].

Feeding strategy is one approach to decrease methane production. This is advantageous for long-term reduction of greenhouse gases and short-term energy loss in ruminants [10]. These conditions have led scientists from different countries to intensively investigate the active compounds found in herbal plants to address the aforementioned issues. In this study, several alternative plants, including *Eurycoma longifolia* (EL), *Cola acuminata* (CLA), *Cassia alata* (CSA), *Cordia obliqua* Auct, *Ceiba pentandra*, *Curcuma longa* Linn, and *Phyllanthus urinaria*, were utilized. Previous studies have reported that herbal plants contain bioactive compounds that can modulate rumen fermentation characteristics, improve digestibility, and suppress enteric methane gas production in livestock [11, 12]. The degradation of feed ingredients in the rumen can be evaluated using several methods, one of which is the *in vitro* method. *In vitro* method is an indirect approach to predict digestibility in the laboratory by mimicking digestive processes in ruminants [13]. Getachew *et al*. [13] added that the *in vitro* method has advantages such as a shorter timeframe, lower cost, and simultaneous processing of many feed samples. In addition, it allows the study of the digestibility of feed ingredients which cannot be given individually to animals.

Previous studies [14, 15] have shown that herbal plants can decrease methane gas emissions. *Nigella sativa*, *Rosmarinus officinalis*, and *Zingiber* are effecctive antimethanogenic tannin-containing herbal plants [16]. Vázquez-Carrillo *et al*. [17] reported that *Cosmos bipinnatum* and *Cymbopogon citratus* decreased the *in vivo* methane production by beef cattle. The use of local herbal plants to reduce methane gas emissions has not yet been widely reported.

This study aimed to use *in vitro* methods to examine how herbal plants could contribute to the reduction of methane gas emissions and to evaluate the effect of potential herbal plant supplementation in the diet on rumen fermentation characteristics, total gas, and methane production, *in vitro* nutrient digestibility, and total protozoa populations within the rumen. This study is particularly needed for ruminants as methane gas emitters contribute to global warming.

## Materials and Methods

### Ethical approval

The Institutional Animal Care and Use Committee, Indonesian Agency for Agricultural Research and Development (IAARD), approved this study protocol (Approval Letter No. Balitbangtan/Lolitkambing/Rm/02/2016).

### Study period and location

This study was conducted from October 2015 to March 2016 at Feed Science and Technology Laboratory of Bogor Agricultural Institute and Food and Nutrition Laboratory of Gadjah Mada University, Indonesia.

### Experiment I

#### Sample collection

In this study, we examined the supplementation of herbal plants to feed, comprising 70% complete ration (palm kernel meal [PKM]) and 30% a mixture of these herbal plants. EL, CLA, CSA*, C. obliqua* Auct, *C. pentandra, C. longa Linn*, and *P. urinaria* were used in this study. These herbal plants were collected from Medical Plant Garden of Materia Medica Batu Herbal Laboratory, East Java, Indonesia. These plants were identified by a botanist from the Materia Medica Batu Herbal Laboratory in East Java, Indonesia. Specimens (*E*. CLA*:* MMB-104Cni and *C. longa* Linn: MMB-268Clo) were placed in the herbarium of Materia Medica Batu Herbal Laboratory, East Jawa, Indonesia. This area has a temperature of 11–19°C and a slope of 7° 52′ S to 112° 31′ E with an altitude of 800-2000 m above sea level. Approximately 6 times a month, the average monthly rainfall is 298 mm.

Leaves were chosen as the experimental sample and ground through a 1 mm sieve after oven-dried for 24 h at 60°C. Experiment I investigated the relationship between the secondary metabolites of all these herbal plants and the *in vitro* evaluation parameters.

#### In vitro method

In Experiment 1, the Theodorou method was employed to evaluate the total gas production and protozoan population of herbal plants [18]. Rumen fluid was obtained from a fistulated Frisian Holstein at the Indonesia Livestock Research Center (Balitnak) Ciawi, Bogor, Indonesia. The rumen fluid was then filtered using a filter cloth, inserted into a flask, and transported to the laboratory. A fermentation tube was filled with 100 ml of rumen fluid, and 0.75 g of the sample and 75 mL of buffer were added to it. The tube was sealed with a rubber cover and incubated for 72 h at 39°C–41°C. We selected three potential herbal plants based on the highest total gas production and the lowest total protozoa production. In Experiment 2, potential herbal plants that demonstrate optimal results will proceed to Experiment 2.

#### Parameter measurement

Total gas and gas production rate

A total of 2, 4, 8, 12, 24, 36, 48, 60, and 72 h after incubation were used to measure the total gas and gas production rate. A plastic syringe with a capacity of 60 mL was used for this measurement. Manual readings were obtained from the scale on the syringe after the gas pushed it perfectly. The total gas volume (measured in mL) was determined [19, 20]. The generated gas pushed the inner part of the syringe upward. As soon as the syringe had been sufficiently displaced by the gas, a manual reading of the scale was recorded. The total gas volume (mL) was then determined. The gas production rate was estimated using the Orskov equation [19].

Total protozoa

A total of 1 ml of the incubation sample was placed in 1 ml of trypan *blue* formalin *saline* (TBFS) solution. The TBFS solution was a mixture of 0.9% physiological NaCl solution and 4% formalin in 100 mL of solution. To determine the population of protozoa, the mixture was dropped into a 0.1-mm counting chamber. The smallest box area was 0.0625 mm^2^, and there were 16 boxes; however, only five boxes were used for reading. A 10× magnification microscope was used to calculate the protozoan population. The following formula was used to calculate the protozoan population:


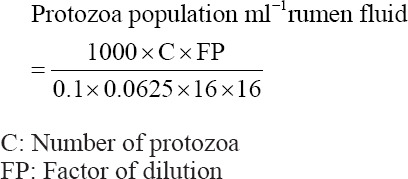


### Experiment II

#### Experimental treatments

This phase focuses on the addition of selected herbal plants to the feed comprising 30% herbal plants and 70% palm kernel cake. According to the results of Experiment 1, EL, CLA, and CSA produced the highest total gas production and suppressed protozoa (Table-1). Experiment 2 used a randomized block design with four replications comprising the following experimental treatments:

**Table-1:** Total gas production and protozoa population of treatments.

Herbal plants	Total gas production (24 h)	Protozoa (Log CFU/mL)
*Eurycoma longifolia*	14.0^d^ ± 1.00	4.11^b^ ± 2.30
*Cassia alata*	27.7^c^ ± 3.55	4.85^a^ ± 2.79
*Ceiba pentandra*	24.8^c^ ± 1.26	4.64^a^ ± 2.63
*Cola acuminata*	80.3^a^ ± 4.54	4.76^a^ ± 2.71
*Cordia obliqua Auct*	4.17^e^ ± 0.58	4.43^a^ ± 2.54
*Curcuma longa Linn*	37.8^b^ ± 2.02	5.00^a^ ± 2.88
*Phyllanthus urinarius*	38.5^b^ ± 0.50	4.85^a^ ± 2.77

Different superscripts a, b, c, d in the same column show significantly different (p *<* 0.05), CFU=Colony-forming unit

T1 : 100% PKM

T2 : 70% PKM + 30% EL

T3 : 70% PKM + 30% CLA

T4 : 70% PKM + 30% CSA

T5 : 70% PKM + 15% EL + 15% CLA

T6 : 70% PKM + 15% EL + 15% CSA

T7 : 70% PKM + 15% CLA + 15% CSA

T8 : 70% PKM + 10% EL + 10% CLA + 10% CSA

In Experiment 2, various parameters, including *in vitro* dry matter digestibility (IVDMD), *in vitro* organic matter digestibility (IVOMD), concentration of NH_3_, pH of the rumen, total volatile fatty acids (VFA), partial VFAs (acetate, propionate, and butyrate), methane gas levels, total gas production, and protozoa population, were measured.

#### Nutrient contents of the sample

Three plants were obtained according to the evaluation process of Experiment I. Bioactive compounds (tannins and saponins) have been identified in the selected plants (Table-2). Herbal plant samples were analyzed for nutrient contents (Table-3) through proximate analysis [21] and analysis of fiber fractions using Goering and Van Soest analysis [22]. Nutrient contents of each treatment are presented in Table-4.

**Table 2:** Bioactive compounds of *Eurycoma longifolia*, *Cola acuminata*, *Cassia alata*.

Parameter	*Eurycoma longifolia*	*Cola acuminata*	*Cassia alata*
Flavanoid	+	+	+
Alkaloid	-	+	-
Tannin	+	+	+
Saponin	+	+	+
Steroid	+	+	+
Triterpenoid	-	-	-
Quinon	-	+	-

**Table 3:** Nutrient contents of feed ingredients.

Nutrient content (%DM)	Feed ingredients			

	Palm kernel meal	*Eurycoma longifolia*	*Cola acuminata*	*Cassia alata*
DM	90.8	90.5	88.0	90.0
Ash	6.61	9.08	6.60	10.5
Crude protein	14.5	8.79	7.95	21.9
Extract ether	6.76	2.16	0.54	3.67
Crude fibre	26.2	30.5	6.90	19.0
NDF	61.7	57.4	45.4	41.2
ADF	39.2	45.5	17.8	27.7
Lignin	10.9	-	-	-
Cellulose	26.3	-	-	-

DM=Dry matter

**Table 4:** Nutrient contents of treatments.

Treatments	Nutrients (%DM)			

	Crude protein	Crude fibre	Extract ether	Ash
T1	14.5	26.2	6.77	6.61
T2	12.8	27.5	5.39	7.35
T3	12.6	20.4	4.63	6.61
T4	16.7	24.0	5.84	7.77
T5	12.7	23.9	5.14	6.98
T6	14.8	25.7	5.61	7.56
T7	14.7	22.2	5.37	7.19
T8	14.1	24.0	5.38	7.25

T1 (100% meal from palm kernel meal), T2 (70% meal from palm kernel meal + 30% *Eurycoma longifolia*), T3 (70% meal derived from palm kernel + 30% *Cola acuminata*), T4 (70% palm kernel meal + 30% *Cassia alata*), T5 (70% palm kernel meal + 15% *Eurycoma* *longifolia +* 15% *Cola acuminata*), T6 (70% palm kernel meal + 15% *Eurycoma* *longifolia* + 15% *Cassia alata*), T7 (70% palm kernel meal + 15% *Cola acuminata* + 15% *Cassia alata*), T8 (70% palm kernel meal + 10% *Eurycoma longifolia* + 10% *Cola acuminata* + 10% *Cassia alata*) DM=Dry matter

#### In vitro method

The Tilley and Terry method was employed to determine organic matter digestibility (IVOMD) and IVDMD [23]. Rumen fluid was obtained from fistulated Friesian Holstein dairy cows and collected for laboratory analysis. Forty milliliters of McDougall’s solution (consisted of NaHCO_3_, Na_2_HPO_4_, KCl, MgSO_4_.7H_2_O, NaCl, and CaCl_2_), 100 mL of rumen fluid [24], and 0.5 g of sample were added to the fermentation tube. To achieve anaerobic conditions, CO_2_ gas flows into the fermentation tube. Afterward, the fermentation tube was enclosed by rubber and aluminum cover, positioned in press, and incubated in a water bath at 39°C for 48 h. After 48 h of incubation, 2–3 drops of HgCl_2_ were added into the fermentation tube. Residues and supernatants were separated using a centrifuge at 1792× *g* for 10 min. The remaining material was filtered through What man paper and dried in an oven at 105°C for 24 h. A porcelain cup was placed in a desiccator and weighed after 24 h to determine the dry matter (DM). The porcelain cup underwent a 6-h firing process in an electric furnace and was then reweighed to determine the organic content. IVDMD and IVOMD were calculated using the formulas outlined by Marlida *et al*. [25].

#### Rumen characteristics determination

The pH of the rumen was assessed using a pH meter (Jenway, USA) initially calibrated with a pH 7 solution before measurement. NH3 concentration was evaluated using Conway modification method [26]. Total and partial VFAs, including acetate, propionate, and butyrate, were measured using a spectrophotometer gas chromatography GC-2010 Plus, No. O205354, Shimadzu, Japan, equipped with a column comprising 10% SP-1200 and 1% H_2_PO_4_ at 80/100 Cromosorb WAW.

#### Methane emission determination

Methane emissions were determined using the Fievez method, in which CO_2_ was trapped using the alkaline capture agent NaOH [27]. Methane gas measurements were conducted after the total gas measurement. As soon as all gas production measurements were completed, methane gas was quantified by disconnecting the syringe attached to the inlet of the 5 M NaOH solution. As soon as the syringe is connected to the NaOH solution inlet, the total gas produced is allowed to pass through the NaOH solution by slowly pushing the syringe. All generated gases were captured by the NaOH solution, while the methane gas component was allowed to pass through the Erlenmeyer outlet. The volume of methane gas (mL) was calculated by manual measurements on the balance.

### Statistical analysis

This study was conducted using a randomized block design. In Experiment 1, the feed ingredient components, total gas production, and gas production rate were evaluated for seven herbal plants, with each sample being repeated 3 times. In Experiment 2, an *in vitro* study with four replications was conducted to evaluate eight treatments. Data from each study were analyzed using analysis of variance with SPSS version 16.0 software. Statistically significant differences (p < 0.05) were examined in more detail using Duncan’s test.

## Results and Discussion

### Experiment 1

#### Total gas production and total protozoa population

Table-1 presents the production of total gas and protozoa for the seven herbal plants. Different herbal plants significantly (p < 0.05) influenced both the amount of gas produced and the number of protozoa present. CLA (80.33 mL), followed by *P. urinaria* (38.50 mL), *C. longa* Linn (37.83 mL), CSA (27.6 mL), *and C. pentandra* (24.83 mL), had the highest total gas contents. *C. obliqua* Auct produced the lowest amount of total gas at 4.17 mL. The results of our research revealed significant differences (p < 0.05) in the protozoan population among herbal plants. EL had the smallest protozoan population compared with the other species. This outcome suggests that the three herbal plants were suitable for testing in Experiment 2, considering the highest total gas production, which was found in CLA. EL exhibited the lowest protozoa population, and CSA stood out when considering herbal plants with an average gas production and protozoa population (Table-1).

Differences were observed in total gas and protozoa production among herbal plants (p <0.05). EL, CSA, *C. pentandra*, *C. longa* Linn, CLA, and *P. urinarius* plants exhibited the highest total gas production, whereas *C. obliqua* Auct had the lowest gas production. EL leaves contain active saponin compounds that suppress protozoan populations. Saponins found in herbaceous plants can enhance the effectiveness of the fermentation process by decreasing protozoa population in the rumen, thereby reducing their predation on bacteria [28]. Supartini and Cahyono [29] also reported that EL leaf extract contains saponins, tannins, carotenoids, triterpenoids, and coumarins, indicating that EL can act as an antiprotozoal agent. According to Tan *et al*. [28], methanogenic bacteria has symbiosis with protozoa. It has been scientifically demonstrated that the removal of protozoa from the rumen (defaunation) leads to a 9–37% reduction in methane emissions. Defaunation reduces the number of methanogens, which are considered to be primary methane producers in the rumen [30].

### Experiment II

#### In vitro evaluation

This research was conducted in Experiment II. EL, CLA, and CSA had the highest production of total gas and the lowest production of protozoa (Table-1), whereas EL had the lowest production of protozoa.

#### In vitro nutrient digestibility

The use of plant-based herbs resulted in a significant increase in IVDMD compared to the control group (p < 0.05) (Table-5). Giving 30% CSA (T4) and a mixture of plant herbs with 15% EL and 15% CLA (T5) at the highest ratio increased IVDMD (Table-5). The decrease in IVDMD after treatment of mixed plant herbs with 10% EL, 10% CLA, and 10% CSA (T8) (Table-5) was comparable to that after treatment with 30% EL, with a value of 63.16% (T2). The DM digestibility of a mixture of herbal plants containing 30% CLA (T3) was 62.84%, which was comparable to the DM digestibility of a mixture of herbal plants containing 15% CLA and 15% CSA (T7) of 62.40%. The DM digestibility of 30% of herbal plants in the ration in this study ranged from 62.40% to 70.51 % (Table-5). The average digestibility of DM was 70.51% and 69.93% in the administration of 30% CSA (T4) and a mixture of plant herbs with *15%* EL *and* 15% CLA. The lowest IVDMD was achieved at an average of 10% EL, 10% CLA, and 10% CSA (T8).

**Table-5:** Feed digestibility and rumen fermentation characteristics of treatments.

Treatments	Feed digestibility		Rumen fermentation characteristics	

	IVDMD (%)	IVOMD (%)	NH_3_ (mm)	pH
T1	41.2^c^ ± 1.71	31.3^e^ ± 1.22	13.9^c^ ± 0.61	6.78 ± 0.08
T2	63.2^b^ ± 2.21	54.4^cd^ ± 1.36	14.4^bc^ ± 2.01	6.73 ± 0.13
T3	62.8^b^ ± 1.49	58.5^b^ ± 2.44	14.1^bc^ ± 3.60	6.73 ± 0.08
T4	70.5^a^ ± 4.52	61.6^a^ ± 4.26	16.7^a^ ± 2.54	6.77 ± 0.03
T5	69.9^ab^ ± 3.64	57.1^bc^ ± 2.38	15.8^abc^ ± 4.21	6.75 ± 0.05
T6	64.5^b^ ± 3.37	57.5^b^ ± 3.35	16.0^a^ ± 3.77	6.78 ± 0.10
T7	62.4^b^ ± 3.24	53.2^d^ ± 3.52	16.7^a^ ± 4.13	6.75 ± 0.05
T8	63.9^b^ ± 1.56	55.7^bcd^ ± 2.49	15.9^abc^ ± 4.74	6.73 ± 0.03

Different superscripts (a, b, c, d) within the same column, point out significant disparities (p *<* 0.05). T1 (comprising entirely palm kernel meal), T2 (70% palm kernel meal + 30% *eurycoma longifolia*), T3 (70% palm kernel meal + 30% *Cola acuminata*), T4 (70% palm kernel meal + 30% *Cassia alata*), T5 (70% palm kernel meal + 15% *eurycoma* *longifolia +* 15% *Cola acuminata*), T6 (70% palm kernel meal + 15% *Eurycoma* *longifolia* + 15% *Cassia alata*), T7 (70% palm kernel meal + 15% *Cola acuminata* + 15% *Cassia alata*), T8 (70% palm kernel meal + 10% *eurycoma longifolia* + 10% *Cola acuminata* + 10% *Cassia alata*), IVDMD*=in vitro* dry matter digestibility, IVOMD=*in vitro* organic matter digestibility

An increase in IVDMD is positively correlated with IVODM. Treatment with herbal plants in the ration led to a notable increase in IVODM compared with the control group (p < 0.05) (Table-5). The largest IVODM was observed in T4 (supplementation 30% CSA*)*. Herbs can enhance the organic digestibility of high-fiber feeds. In this study, the breakdown of organic matter in the herbal plant treatment varied between 53.18% and 61.61% (Table-5).

The introduction of herbal plants significantly enhanced IVDMD compared with the control group (p < 0.05; Table-5). Our findings indicate that herbal plants contain a high level of degradable protein, which is essential for rumen microbial activities. It is believed that these nutrients promote the growth and activity of rumen microbes and enhance the digestion of the feed compared to the treatment without herbal supplements. This outcome aligns with findings from an earlier investigation that supplementation of herbal plants, such as onions in rations, can increase IVDMD and IVOMD values compared with control [31]. This study demonstrated that supplementation with 30% CSA can increase IVDMD and IVOMD. The presence of saponins, flavonoids, tannins, alkaloids, and phenolic compounds in CSA effectively inhibits the activity of harmful microorganisms. The growth of pathogenic microorganisms is inhibited, allowing non-pathogenic microbes to work more optimally, resulting in an increase in nutrient digestibility [32].

The increase in IVDMD was positively correlated with IVODM (Table-5). Herbal plants can enhance organic matter digestibility in high-fiber feeds. The findings of our study indicated that herbal plants had no impact on microbial activity during rumen fermentation process, thereby not affecting the digestibility of DM and organic matter in the feed ingredients. This finding is consistent with the study by Muchlas *et al*. [33], in which the inclusion of herbal plants in rations resulted in an IVODM range of 61.61%–53.18%. This shows that herbal plants do not interfere with rumen microorganisms involved in the digestion of organic matter. The supplementation with herbs did not reduce the nutrient digestibility. This shows that herbal plants do not disrupt the activity of rumen microbes in digesting organic matter. A previous study reported that supplementation with herbal plants, such as onions, increased IVDMD and IVODM values compared with controls: 64.1% versus 62% for IVDMD and 62.3% versus 59.6% for IVODM [33].

#### Rumen fermentation characteristics

The administration of herbal plants improved the NH_3_ concentrations (p < 0.05) (Table-5). NH_3_ concentration in this study ranged from 13 to 17 mM. The highest concentration (16.67 mM) was found in the administration of 30% CSA (T4). Meanwhile, T8 had the same result as the T5 treatment and was not much different compared with the T2 and T3 treatments. T1 had the lowest value, 13.85 mM, observed in the treatment without herbs. However, T4, T6, and T7 treatment showed almost the same NH_3_ concentrations (Table-5). The rumen pH values during the incubation of the eight feed treatments ranged from 6.73 to 6.78, and there was no significant difference (p > 00.05) (Table-5). Feeding herbal plants at 30% does not interfere with rumen environmental conditions, such as the normal pH of the rumen. *In vitro* rumen pH values of 6.73–6.78 are within the normal range (Table-5).

No statistically significant difference was observed between the total and partial VFA levels in this study, including acetate, propionate, and butyrate (p > 0.05) (Table-6). The total VFA values in this study varied between 34.28 and 45.43 mM. The T4 treatment containing 30% CSA exhibited the highest total VFA concentration, whereas the T8 treatment containing 10% EL, 10% CLA, and 10% CSA exhibited the lowest concentration.

**Table 6:** VFA concentrations of treatments.

Treatments	Acetate (%)	Propionate (%)	Butyrate (%)	VFA total (mM)
T1	62.0 ± 3.34	22.3 ± 0.75	15.7 ± 2.63	34.8 ± 7.94
T2	62.6 ± 2.95	22.1 ± 1.39	15.3 ± 2.06	38.2 ± 8.19
T3	62.0 ± 2.82	21.9 ± 0.45	16.1 ± 2.83	36.5 ± 6.95
T4	63.1 ± 2.46	21.6 ± 1.68	15.3 ± 1.76	45.4 ± 14.0
T5	63.4 ± 2.33	21.7 ± 0.77	14.8 ± 2.08	37.8 ± 11.0
T6	63.1 ± 2.30	21.8 ± 0.48	15.1 ± 2.77	35.6 ± 4.27
T7	62.9 ± 1.53	21.6 ± 0.63	15.5 ± 2.01	38.3 ± 4.43
T8	63.2 ± 2.96	21.6 ± 0.58	15.3 ± 2.62	34.3 ± 3.94

VFA=Volatile fatty acid. T1 (palm kernel meal at 100%), T2 (70% palm kernel meal + 30% *Eurycoma longifolia*), T3 (70% palm kernel meal + 30% *Cola acuminata*), T4 (70% palm kernel meal + 30% *Cassia alata*), T5 (70% palm kernel meal + 15% *Eurycoma* *longifolia +* 15% *Cola acuminata*), T6 (70% palm kernel meal + 15% *Eurycoma* *longifolia* + 15% *Cassia alata*), T7 (70% palm kernel meal + 15% *Cola acuminata* + 15% *Cassia alata*), T8 (70% palm kernel meal + 10% *Eurycoma longifolia* + 10% *Cola acuminata* + 10% *Cassia alata*)

The optimal functioning of rumen microbes depends on the pH of the rumen and the current environmental conditions. Maintaining normal rumen metabolism requires a normal rumen pH ranging from 6.0 to 7.0. Rumen pH can affect the population of microorganisms active in fermentation process [34]. The rumen pH values of the eight treatments ranged from 6.73 to 6.78. Rumen pH is still in the normal range. According to a previous study, the normal pH range of rumen is 5.4–7.8. Rumen pH is influenced by feed quality and microbial fermentation processes in the rumen [35, 36]. Extreme increases or decreases in pH values in the rumen impact the high mortality of microorganisms in the rumen [37]. Our findings indicate that herbal plants do not affect the pH of the rumen, allowing the rumen fermentation process to proceed optimally.

NH_3_ (ammonia) can be measured to determine the source of nitrogen in the rumen. Rumen microorganisms use ammonia for microbial protein synthesis [38-40]. Rumen microbes perform microbial protein synthesis from NH_3_, which comes from the deamination of amino acids and the hydrolysis of nonprotein nitrogen, which serves as the main precursor for protein synthesis in ruminants [41-42]. In the present study, we showed that the addition of herbal plants can enhance the activity of microbes to degrade protein in the rumen of ruminants, leading to an increase in the ammonia concentration in the rumen. Increased protein breakdown increases the production of NH3 in the rumen. The source of rumen N–NH_3_ is the breakdown of protein in the feed and microbial protoplasm, particularly protozoa. Protozoa play a role in regulating the provision of soluble protein to support the proliferation of bacteria [38]. The range of NH_3_ (ammonia) in this study corresponds to the ideal ammonia range needed in the rumen (85–300 mg/l or 6–21 mM, respectively) [43]. The ammonia content in the rumen is the result of the degradation activity of protein content and endogenous proteins by rumen microorganisms through the N balance mechanism in ruminants [43.^44^].

Total VFA indicates the amount of feed fermented by rumen microbes [45]. Acetate is produced in large quantities (approximately 20–50 mol/day), and propionate accounts for one-third of the production. Butyrate accounts for approximately 10% of the total acid produced, and valeric and isovaleric acids account for 1%–2% [46]. There was no significant difference (p *=* 0.05) in the total VFA concentrations. According to McDonald *et al*. [47], the total concentration of VFA ranged from 70 mM to 150 mM. Total VFA indicates the amount of organic matter in the feed that can be easily broken down by rumen microorganisms. Maintaining a balanced rumen pH is essential for the proper functioning of rumen microorganisms, which is linked to higher VFA concentration. The availability of feed ingredients for microbial fermentation increases as the incubation time increases. As a result, there is a decrease in the production of VFA, indicating reduced energy availability for ruminants.

Several factors influence the level of VFA concentration, including the type of carbohydrates dissolved, the physical form of the feed, rumen pH, digestibility, and the addition of additional chemicals to the feed [48]. However, there were no significant differences in partial concentrations, including acetate, propionate, and butyrate (p < 0.05). The ratio composition partly affects the VFA. The production of propionic, acetic, and butyric acids is mainly based on carbohydrate fermentation and, to a lesser extent, on protein fermentation. Saponins present in herbal plants can enhance rumen bacterial count, overall VFA, and acetate, propionate, and NH_3_ concentrations. Acetate, propionate, and butyrate generate H_2_ and CO_2_, respectively, which methanogenic bacteria use to produce CH_4_ (Table-6).

#### Total gas and rate of gas production

A notable variance in total gas production was attributed to the presence of herbal plants (p < 0.05; Table-7). Treatments with EL, CLA, CSA, or a mixture of these compounds enhanced the production of the entire gas. Treatment T3 showed the highest total gas, whereas treatment T2 with EL resulted in the lowest. The combination treatment of EL + CLA + CSA (T8) yielded higher results than T7, which had only two plant combinations: CLA + CSA. There was no significant difference (p >00.05) in the gas production rate between the treatments.

**Table-7:** Total gas and rate of gas production of treatments.

Treatments	Total gas (mL)	Gas production rate (mL/h)
T1	109^e^ ± 7.25	0.052 ± 0.003
T2	115^de^ ± 3.71	0.048 ± 0.002
T3	148^a^ ± 7.16	0.051 ± 0.003
T4	124^c^ ± 10.0	0.049 ± 0.001
T5	119^cd^ ± 11.2	0.050 ± 0.005
T6	124^c^ ± 6.22	0.051 ± 0.004
T7	132^b^ ± 8.52	0.050 ± 0.003
T8	133^b^ ± 7.46	0.049 ± 0.003

Distinct superscripts (a, b, c, d) within the identical column indicate significant disparities (p *<* 0.05). T1 (entire palm kernel meal), T2 (70% palm kernel meal + 30% *Eurycoma longifolia*), T3 (70% palm kernel meal + 30% *Cola acuminata*), T4 (70% palm kernel meal + 30% *Cassia alata*), T5 (70% palm kernel meal + 15% *Eurycoma* *longifolia +* 15% *Cola acuminata*), T6 (70% palm kernel meal + 15% *Eurycoma* *longifolia* + 15% *Cassia alata*), T7 (70% palm kernel meal + 15% *Cola acuminata* + 15% *Cassia alata*), T8 (70% palm kernel meal + 10% *Eurycoma longifolia* + 10% *Cola acuminata* + 10% *Cassia alata*)

Our findings demonstrate a substantial impact (p < 0.05) of herbal plant supplementation on total gas production (Table-7). These results indicate that EL, CLA, and CSA, or a combination of both, can enhance gas production compared with the control group. Herbal plants can stimulate the activity of microorganisms in the rumen, which in turn affects the fermentation of feed nutrients. An increase in fermentation process leads to higher gas production [20]. The different doses of herbal plants in the ration have a significant impact on total gas production. The T3 treatment (CLA substitution) resulted in the highest total gas. The highest total gas production in T3 was due to feed degradation in the rumen. The lowest extract ether level in T3 (Table-4) had the fastest feed degradability and the highest total gas production. In addition, CLA exhibits high degradability due to its highest total gas production (Table-1). Therefore, CLA supplementation in the diet resulted in the highest total gas production. In line with Kannan *et al*. [49], which indicate that gas production relies on both the chemical composition and degradability of the feed.

Measuring total gas *in vitro* can be used to detect the decomposition of organic matter, particularly nitrogen and carbon [20, 25]. The increase in total gas produced when herbal plants are combined shows that it does not affect microbial activity in gas production. Active substances in herbal plants do not inhibit the growth or activity of rumen microbes [10].

#### Methane emissions and total protozoa population

Supplementation with herbal plants or their combination (T2, T3, T4, T6, T7, and T8) had a significantly different effect on methane gas decrease (p < 0.05; Table-8). Methane gas production was the highest in treatment T1 (control) and lowest in treatment T2. The lowest methane gas emissions were observed in the T2 (30% EL) and T5 (15% EL + 15% CLA) treatments. This study demonstrated that supplementing herbal plants or their combinations (T2, T3, T4, T6, T7, and T8) did not significantly reduce total protozoan populations (p > 0.05) (Table-8).

**Table-8:** The methane gas production and total protozoa of treatments.

Treatments	Methane gas production (mL methane/g OMD)	Total protozoa (Log CFU/mL)
T1	134^e^ ± 17.7	4.71 ± 0.079
T2	100^a^ ± 21.2	4.46 ± 1.016
T3	109^b^ ± 19.0	4.50 ± 0.610
T4	104^bc^ ± 17.5	4.39 ± 0.525
T5	101^a^ ± 13.6	4.18 ± 0.087
T6	116^c^ ± 11.6	4.81 ± 0.022
T7	122^cd^ ± 16.7	4.40 ± 0.519
T8	126^d^ ± 19.3	4.42 ± 0.400

Different superscripts a, b, c, d within the identical column indicate remarkable distinctions (p *<* 0.05). T1 (entire palm kernel meal), T2 (70% palm kernel meal + 30% *Eurycoma longifolia*), T3 (70% palm kernel meal + 30% *Cola acuminata*), T4 (70% palm kernel meal + 30% *Cassia alata*), T5 (70% palm kernel meal + 15% *Eurycoma* *longifolia +* 15% *Cola acuminata*), T6 (70% palm kernel meal + 15% *Eurycoma* *longifolia* + 15% *Cassia alata*), T7 (70% palm kernel meal + 15% *Cola acuminata* + 15% *Cassia alata*), T8 (70% palm kernel meal + 10% *Eurycoma longifolia* + 10% *Cola acuminata* + 10% *Cassia alata*), CFU=Colony-forming unit

A significant impact of supplementation with herbal plants or their combinations in reducing methane gas was observed (p < 0.05) (Table-8). Maximum methane gas production was observed in treatment T1 (control), whereas the lowest production was observed in treatment T2. Methane is produced by methanogens during the anaerobic fermentation process of the ration in the digestive tract of the rumen, reflecting a loss of energy from the feed. According to Mcdonald *et al*. [47], a higher production of CH_4_ gas implies less efficient energy use. Herbal plants contain a variety of compounds, such as phenolic compounds, saponins, tannins, alkaloids, and flavonoids. Saponin affects the properties of the rumen and reduces methane gas emissions, which is positively correlated with the number of methanogens and protozoa. These compounds inhibit the activity of pathogenic microbes, such as methanogens.

Direct reduction of methanogenic bacteria or methane-producing bacteria reduces methane gas production [11]. There was a significant difference in methane gas concentration among treatments with either a single herbal plant (p < 0.05) (Table-8). Differences in the concentration of compounds in various herbal plants can cause variations in the active substances found in a single herb or a combination of herbs, which may explain our findings. The addition of one herbal plant or a combination did not significantly reduce the protozoan population (Table-8). The combination of EL with CLA resulted in the lowest protozoan population compared with the other treatments. The decrease in protozoa populations in EL and CLA may be caused by saponins [28, 29]. Saponins form bonds with sterols found in protozoan cell walls, thereby influencing the surface tension of protozoan cell membranes.

Compounds present in herbal plants, such as saponins and tannins, increase cell wall permeability, resulting in fluid entering the protozoan cell. The entry of fluids from outside the cell leads to damage to the cell wall, resulting in death or lysis. Tannins can bind proteins in the rumen, leading to a lack of nutrients for protozoa to grow. Tannin compounds from earth bolts and cola can also damage protozoan rumen cell membranes [50, 51]. The combination of the two compounds has a good effect in reducing the protozoan population, thereby optimizing the functioning of the rumen microbe.

## Conclusion

EL, CLA, and CSA have the potential to reduce enteric methane and increase *in vitro* nutrient digestibility and NH_3_ and VFA production. The combination of EL and CLA yielded the best results in reducing methane gas and total protozoan concentrations in rumen fermentation. Based on the evaluation results of several herbal plants, singly and in combination, with the use of 70% PKM and 30% CSA content in the *in vitro* methods, a higher percentage of NH_3_ and nutrient digestibility were observed, which is easier to degrade than CLA and EL. When 30% CLA is added, the highest total gas production occurs. A combination of 70% PKM, 15% EL, and 15% CLA yielded the best results in reducing methane gas and total protozoa in rumen fermentation. This research was conducted using *in vitro* treatment; therefore, an in vivo approach is needed to test the efficacy of using herbal plants. Extraction and use of active compounds from the three plants as feed additives is an interesting research topic that can be conducted in the future.

## Author’s Contributions

All authors have contributed to the formulation and conceptualization of an experimental design. AA and MIA: Performed the experiment in the laboratory. AA, RP, and MIA: Collected the data in the laboratory. DD, EBL, AJ, LM, and SA: Analyzed the data and supervised the study. AA, RP, EMP, LRA, RDP, RAG, WN, and PSN: Wrote the original and finalized the manuscript. All authors have read, reviewed, and approved the final manuscript.
